# Atrial Septal Defect in a Young Boy Presenting With a Rare Crochetage Sign: A Case Report

**DOI:** 10.7759/cureus.77373

**Published:** 2025-01-13

**Authors:** Abdullah Al Faisal, Kainat Ferdous, Harsimran Kaur Surinder Singh, Faisal Ahmed

**Affiliations:** 1 Emergency Medicine, Hull University Teaching Hospitals NHS Trust, Hull, GBR; 2 Acute Medicine, Hull University Teaching Hospitals NHS Trust, Hull, GBR; 3 Surgery, Hull University Teaching Hospitals NHS Trust, Hull, GBR; 4 Cardiology, National Institute of Cardiovascular Diseases, Dhaka, BGD

**Keywords:** atrial septal defect (asd), crochetage sign, echocardiography, electrocardiogram (ecg), pulmonary hypertension (ph)

## Abstract

An atrial septal defect (ASD) is a common congenital heart anomaly that results in irregular blood flow between the systemic and pulmonary circulations due to an opening in the atrial septum. Ostium secondum ASD accounts for a large proportion of these defects and often goes unnoticed during childhood and adolescence. Pulmonary hypertension (PH), affecting a significant number of patients with ostium secondum ASD, is associated with functional limitations, heart failure, and tachyarrhythmias.

This case involves a 23-year-old male with a large ostium secondum ASD, moderate PH, and normal left ventricular systolic function, presenting with a rare Crochetage sign on his electrocardiogram (ECG). The patient's clinical presentation, including the presence of the Crochetage sign, was assessed using various diagnostic tools. An ECG identified the characteristic notch in the R wave in the inferior leads, indicative of ASD. Further evaluation, including echocardiography, was performed to assess the size and impact of the ostium secondum ASD. Echocardiography confirmed the presence of a large ASD with moderate PH. The case underscores the importance of recognizing rare signs, like the Crochetage sign, in the diagnosis and management of ASD. It presents a clinical dilemma regarding whether to proceed with the closure of the ostium secondum, given the potential complications associated with complete closure. A multidimensional approach, considering the patient's overall condition and potential risks, is essential for optimal management. This case highlights the need for comprehensive evaluation and timely intervention to improve outcomes in patients with ASD.

## Introduction

An atrial septal defect (ASD) is a congenital heart abnormality marked by a hole in the atrial septum, which permits blood to flow between the left and right atria. This case study presents a 23-year-old male patient with a history of progressive shortness of breath, palpitations, and fever, ultimately diagnosed with ASD.

Early diagnosis of ASD can be facilitated by recognizing specific signs on an electrocardiogram (ECG). One such sign is the Crochetage sign, which is a distinctive notch near the apex of the R wave in the inferior leads (II, III, and aVF) [1]. This sign is highly specific for ASD, particularly the ostium secundum type, and its presence can significantly aid in the early detection and management of the condition. In this case, the patient exhibited the Crochetage sign, which contributed to the timely diagnosis of ASD.

## Case presentation

A 23-year-old male patient presented with shortness of breath for one year, palpitations for six months, and fever for two months. The patient was in good health until a year ago, when he began experiencing progressive breathlessness, initially during moderate to severe exertion and relieved by rest. Over the past month, the shortness of breath has been triggered by mild exertion. He also reported occasional palpitations during exertion, which were relieved by rest. There were no symptoms of respiratory distress at rest, chest pain, or significant weight loss.

The patient had no history of hypertension, diabetes, asthma, or rheumatic fever. He was a non-smoker, non-alcoholic, and had no history of drug abuse. There was no family history of similar illness, and he belonged to a low socioeconomic background.

On physical examination, the patient appeared ill-looking but cooperative, with a BMI of 18.5 kg/m². Vital signs included a pulse of 80 bpm, BP of 110/80 mmHg, respiratory rate of 18 breaths/min, temperature of 98°F, and oxygen saturation of 99%. Cardiovascular examination revealed normal heart sounds, wide fixed splitting of the second heart sound, and a systolic murmur in the left upper intercostal space. Respiratory examination showed clear lung bases with no added sounds, and other systems revealed no abnormalities.

**Figure 1:**
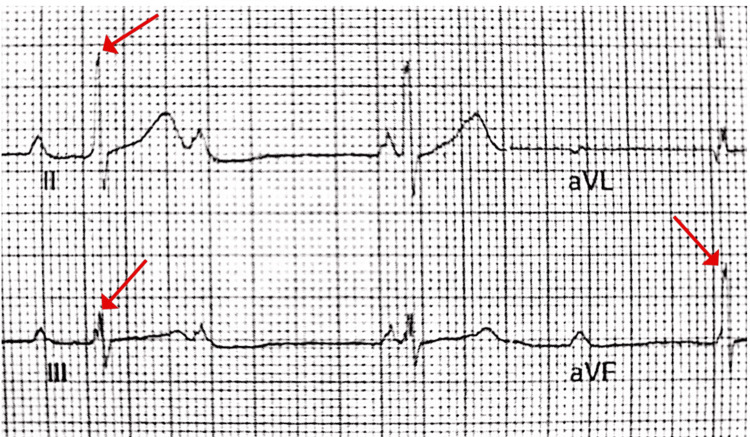
ECG finding consistent with Crochetage sign (a notch located near the peak of the R wave in the inferior leads (II, III, aVF)) The red arrows indicate notched R waves. Image credit: [2]; this is an open-access article distributed under the terms of the Creative Commons Attribution License CC-BY 4.0., which permits unrestricted use, distribution, and reproduction in any medium, provided the original author and source are credited. ECG, electrocardiogram

Diagnostic workup included a complete blood count (CBC) within normal limits, an ECG showing sinus rhythm, right bundle branch block (RBBB), right axis deviation, right ventricular hypertrophy (RVH), and the Crochetage sign, which helped to diagnose the case early (Figure 1). A chest X-ray indicated cardiomegaly, dilated pulmonary vessels, and plethoric lung fields. Echocardiography revealed a large ASD secundum, moderate pulmonary hypertension (PH), and normal left ventricular systolic function (LVEF = 66%).

The patient was diagnosed with ostium secundum type of ASD with significant left-to-right shunt and moderate PH. The treatment plan included medical management with beta-blockers and diuretics, and surgical management was recommended for ASD closure due to evidence of right ventricular volume overload and significant left-to-right shunt. The ASD was successfully corrected with percutaneous device closure, and the Crochetage sign subsequently disappeared.

## Discussion

An ASD is a common congenital heart condition in adults. Clinically, ASD is characterized by a wide and fixed split of the second heart sound and an ejection systolic murmur at the upper left sternal border, which is confirmed via echocardiography.

In 1996, Heller et al. identified the Crochetage pattern of the R wave in the inferior limb leads as commonly seen in patients with ASD, correlating with the severity of the shunt and independent of the RBBB pattern. Their retrospective study observed this pattern in at least one inferior lead in 73% of patients with a secundum ASD and 36% of patients with a ventricular septal defect (VSD), compared to only 7% of the general population. The specificity for diagnosing ASD increased to 92%-100% when the pattern was associated with an incomplete RBBB or was present in all three inferior leads [3].

The occurrence of the Crochetage sign in ASD rises with larger anatomical defects or increased left-to-right shunts. In 1998, Ay et al. found that the presence of the Crochetage pattern on an ECG could aid in identifying stroke patients with a patent foramen ovale (PFO), streamline their diagnostic process, and suggest the need for future research to evaluate its effectiveness. The Crochetage sign, reminiscent of the intricacies of crochet needlework, is marked by a notch near the peak of the R wave in the inferior leads (II, III, and aVF). This sign is particularly specific for diagnosing ostium secundum ASD, with its specificity rising to 92% when present in all inferior leads. The Crochetage sign operates independently of the incomplete RBBB pattern, yet when RBBB coexists with Crochetage in all inferior limb leads, the specificity of the ECG diagnosis for ASD is exceptionally high. After surgery, this pattern tends to disappear early in approximately one-third of patients, stratifying stroke risk in PFO patients. The sensitivity and specificity of the Crochetage pattern for diagnosing PFO in cases of cryptogenic stroke were 36% and 91%, respectively, with a positive predictive value of 77% [4].

However, interpreting clinical signs and ECG findings can be challenging, particularly in ICU settings where patients may be restless and uncooperative. In such scenarios, a bedside ECG can provide critical diagnostic clues, with the Crochetage sign being particularly significant [5]. It closely aligns with the extent of left-to-right shunting and the size of the ASD.

The Crochetage sign, reminiscent of the intricacies of crochet needlework, is marked by a notch near the peak of the R wave in the inferior leads (II, III, and aVF). This sign is particularly specific for diagnosing ostium secundum ASD, with its specificity rising to 92% when present in all inferior leads. The Crochetage sign operates independently of the incomplete RBBB pattern, yet when RBBB coexists with Crochetage in all inferior limb leads, the specificity of the ECG diagnosis for ASD is exceptionally high. After surgery, this pattern tends to disappear early in approximately one-third of patients [6].

A retrospective study categorized participants into two groups depending on whether the Crochetage sign was present or absent. The Crochetage sign was characterized by an M-shaped or bifid pattern notch on the R wave in one or more of the inferior limb leads. This sign could suggest prolonged hemodynamic stress and may also help predict the likelihood of late atrial arrhythmias after percutaneous ASD closure [7].

The Crochetage pattern, an 'M'-shaped notch on the R wave in inferior leads, is linked to atrial shunts like ASD and PFO. It helps identify PFOs in cryptogenic stroke patients and indicates higher risks of decompression sickness in divers with PFO [8]. Research shows a correlation between larger infarct sizes and the presence of crochetage in PFO patients, with a significant prevalence in those with cryptogenic stroke. The pattern's high specificity and positive predictive value make it a useful tool for detecting paradoxical emboli. Overall, ECGs with crochetage can expedite the diagnostic process for PFOs, especially in divers with recurrent decompression illnesses.

**Figure 2:**
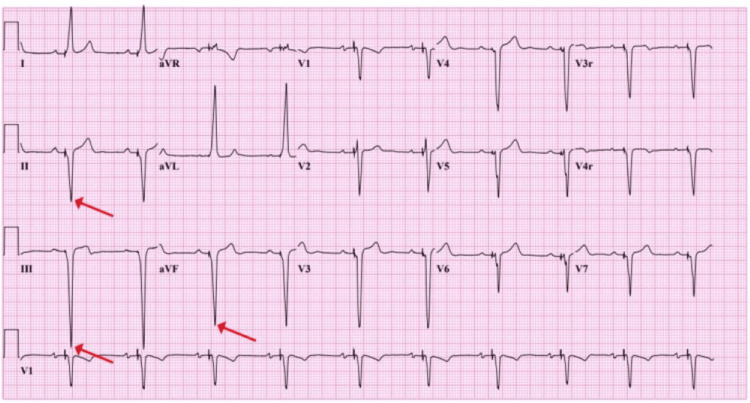
Disappearance of Crochetage sign post-PFO closure The red arrows indicate the disappearance of the notch in the QRS complex*.* Image credit: [2]; this is an open-access article distributed under the terms of the Creative Commons Attribution License CC-BY 4.0., which permits unrestricted use, distribution, and reproduction in any medium, provided the original author and source are credited. PFO, patent foramen ovale

The Crochetage sign diminishes or vanishes after PFO closure, likely due to the elimination of the right-to-left shunt found in patients with PFO, resulting in a more uniform and synchronous depolarization of the right and left ventricles [8]. Consequently, the reduction or disappearance of the Crochetage sign post-PFO closure can be a valuable indicator of the procedure's success (Figure 2).

In summary, the Crochetage sign is a valuable diagnostic tool for ASD, especially in challenging clinical settings. Its presence on an ECG can provide important diagnostic clues, facilitate early diagnosis, and guide appropriate management, ultimately improving patient outcomes.

## Conclusions

This case underscores the critical role of early and accurate diagnosis in managing ASD. The presence of the Crochetage sign on the ECG was pivotal in diagnosing the condition early, highlighting its value as a diagnostic tool, especially in challenging clinical settings. The patient's symptoms, including progressive shortness of breath and palpitations, were effectively correlated with the diagnostic findings, leading to a comprehensive management plan involving both medical and surgical interventions. The timely identification and appropriate management of ASD can prevent complications such as PH and right heart failure, ultimately improving patient outcomes. This case reinforces the need for clinicians to be vigilant in identifying subtle diagnostic clues and underscores the value of integrating clinical findings with advanced diagnostic tools for optimal patient care.
